# Vehicle Logo Recognition Using Spatial Structure Correlation and YOLO-T

**DOI:** 10.3390/s23094313

**Published:** 2023-04-27

**Authors:** Li Song, Weidong Min, Linghua Zhou, Qi Wang, Haoyu Zhao

**Affiliations:** 1 School of Software, Nanchang University, Nanchang 330047, China; 2 School of Mathematics and Computer Science, Nanchang University, Nanchang 330031, China; 3 Institute of Metaverse, Nanchang University, Nanchang 330031, China; 4 Jiangxi Key Laboratory of Smart City, Nanchang 330031, China; 5 School of Information Engineering, Nanchang University, Nanchang 330031, China

**Keywords:** YOLO-T, vehicle logo detection, spatial structural correlation, background interference

## Abstract

The vehicle logo contains the vehicle’s identity information, so vehicle logo detection (VLD) technology has extremely important significance. Although the VLD field has been studied for many years, the detection task is still difficult due to the small size of the vehicle logo and the background interference problem. To solve these problems, this paper proposes a method of VLD based on the YOLO-T model and the correlation of the vehicle space structure. Aiming at the small size of the vehicle logo, we propose a vehicle logo detection network called YOLO-T. It integrates multiple receptive fields and establishes a multi-scale detection structure suitable for VLD tasks. In addition, we design an effective pre-training strategy to improve the detection accuracy of YOLO-T. Aiming at the background interference, we use the position correlation between the vehicle lights and the vehicle logo to extract the region of interest of the vehicle logo. This measure not only reduces the search area but also weakens the background interference. We have labeled a new vehicle logo dataset named LOGO-17, which contains 17 different categories of vehicle logos. The experimental results show that our proposed method achieves high detection accuracy and outperforms the existing vehicle logo detection methods.

## 1. Introduction

In recent years, there has been a significant increase in the number of vehicles on the roads. However, this rise in the number of vehicles not only poses safety hazards but also exacerbates traffic congestion and other issues. As such, it is imperative to construct a modern intelligent transportation system (ITS) [1,2,3]. A fundamental component of the ITS system construction process is the development of vehicle logo detection technology (VLD). In a real-world environment, there are vast numbers of vehicles with a wide range of models. Nonetheless, the vehicle logo is a characteristic attribute that is shared by vehicles of the same brand and holds information related to identity. This information can prove useful in effectively identifying the manufacturer of a vehicle from a large number of vehicle families.

To promote the development of VLD technology, many researchers have conducted extensive research in this area [4,5]. Unfortunately, most of the existing VLD methods are not robust and cannot maintain good detection accuracy when faced with environmental interference. Therefore, VLD remains a challenging task that warrants further research. Nonetheless, VLD is a task of great practical significance as it aims to extract vital identity information about a vehicle. Currently, obtaining vehicle information is primarily achieved using license plate detection technology [6,7,8] and vehicle-type recognition methods [9,10,11]. However, the vehicle logo is often overlooked as an essential component in this process. In fact, the vehicle logo contains a wealth of information and has clear advantages that other vehicle components may lack. Compared with license plates and vehicle models, vehicle logos are simpler and possess unique shapes, making them easier to remember and recognize. Consequently, it is evident that vehicle logos are critical and research on VLD technology is necessary. It should be noted that vehicle logos are not independent components but are instead inherently linked to the vehicle itself. False detection is an unavoidable issue when performing VLD tasks in a highly complex background that includes advertisements and text, among other elements. As such, it is crucial to identify the correlation between the logo and the vehicle, which can effectively solve the problem of false detection of the vehicle logo. In addition, deep learning is applied to the crack digital image classification, such as Yun Que et al. [12] who proposed a crack digital image enhancement method based on a generative adversarial network and an improved deep learning network for asphalt pavement cracks classification.

In previous studies, researchers have widely used the positional correlation between license plates and vehicle logos to locate the latter. These methods involve detecting and locating the license plate first, then using prior knowledge about the relationship between the license plate and the vehicle logo to determine the location of the latter. The next step involves extracting the features of the vehicle logo to achieve recognition. Unfortunately, the positional relationship between license plates and vehicle logos is not constant, which renders these methods less robust. The complexity of the environment may lead to situations where license plates are removed or obscured, which makes it difficult to locate the vehicle logo using these methods. When the license plate is removed or blocked, the existing method cannot effectively extract the region of interest containing the vehicle logo. The position relationship between the license plate and vehicle logo is not simple, and the existing method is difficult to ensure the accuracy of the area of interest. Under such circumstances, such methods will completely fail. In the task of vehicle logo detection, there are many existing difficulties. Vehicles are usually in a complex traffic scene, so many background factors may cause false detection and affect the accuracy of the VLD task. In addition, many superior object detection methods cannot be directly applied due to the lack of labeled vehicle logo datasets. All of the above are problems that need to be solved in the field of VLD. To solve these problems, we have labeled a vehicle logo dataset named LOGO-17 and proposed a new VLD method. The LOGO-17 contains 18,089 images of various scenes from 17 different types of vehicle logos. To promote the development of VLD, this paper proposes a method of VLD based on the YOLO-T model and the correlation of the vehicle space structure. Our method has achieved good VLD accuracy and solved the problem of false detection caused by scene factors. Our contributions are as follows:

1. Aiming at the small size of the vehicle logo, we propose a vehicle logo detection network called YOLO-T. It integrates multiple receptive fields and establishes a multi-scale detection structure suitable for VLD tasks;
2. Aiming at the background interference, we use the position correlation between the vehicle lights and the vehicle logo to extract the region of interest of the vehicle logo;
3. A novel dataset is constructed named LOGO-17, which contains 17 different categories of vehicle logos.

The rest of this paper is organized as follows. Section 2 provides an overview of the work related to the field of VLD. Section 3 detained describes our method of VLD. The experimental results are shown in Section 4. The conclusion and future works are discussed in Section 5.

## 2. Related Works

Up to now, researchers have been cultivating the field of vehicle logo detection for several years and have proposed numerous methods. These methods can be broadly classified into two main categories: traditional methods and deep learning methods, even though they may differ from one another.

The traditional method mainly extracts vehicle logo features through traditional image processing technology to achieve the purpose of detecting vehicle logos. Mao et al. [13] proposed a VLD method based on filtering. They used horizontal and vertical filters to generate two new images and derived a salient image from each of these images. Then they create a binary image from the exported salient images and locate the vehicle logo. Peng et al. [14] applied a method based on the statistical random sparse distribution (SRSD) features to the task of vehicle logo detection. This method can achieve better accuracy but the disadvantage is that it is not robust enough. Psyllos et al. [15] introduced the scale-invariant feature transform (SIFT) technology to the task of vehicle logo detection. This method has achieved good results but it is easily affected by complex environments. Zhao et al. [16] first use prior knowledge to roughly locate the car logo, and then accurately locate the car logo. Yu et al. [17] introduced the overlap enhancement mode of directional edge amplitude into the VLD task. Abdallah et al. [18] proposed a vehicle logo classification method using a two-dimensional principal component analysis and support vector machine ensemble but the effect is not well. Chen et al. [19] proposed a method that combines spatial scale-invariant feature transform (SIFT) with logistic regression classifier to accomplish the vehicle logo recognition task. In [20], the author introduced the directional gradient histogram and support vector machine technology into the vehicle logo detection task and achieved good results. In reference [21], the authors proposed a step-by-step vehicle logo recognition method. They first locate the general position of the vehicle logo and then use image matching and texture features to identify the vehicle logo. Zhao et al. [22] proposed a vehicle sign detection method based on saliency detection. Their method does not require the use of the spatial relationship between license plates and vehicle logos. Du et al. [23] proposed a step-by-step vehicle logo detection method. They first locate the vehicle logo based on the location information of the license plate and then use SIFT representation and SVM to classify the vehicle logo. In reference [24], the author introduced a vehicle identification method based on a dimensionality reduction SIFT vector. This method reduces the amount of calculation by applying dimensionality reduction to the SIFT feature vector. Li et al. [25] introduced edge detection and morphological filtering into the positioning task of vehicle logos. Nie et al. [26] realize vehicle logo recognition using the technology based on the foreground-background pixel pair feature. They proved that the recognition method based on foreground-background pixel pair (FBPP) features has higher recognition performance than the method based on features that mainly focus on gray information. Gu et al. [27] proposed a multi-scale vehicle identification method. This method introduces the directional SIFT stream parsing technology; however, this method relies too much on the license plate. Soon et al. [28] introduced the moment invariant and the minimum mean distance classifier technology into the task of VLD and verified the detection effect of the method on a dataset containing six different types of vehicle logos. Lu et al. [29] proposed a size-self-adaptive vehicle logo recognition method, which uses feature extraction and an SVM classifier. In reference [30], the authors introduced moderate AdaBoost and radial Tchebichef moments into the task of VLD, which overcomes the problems of viewpoint variation and non-symmetric front-license-plate location.

The deep learning method is to use the convolutional neural network to learn the vehicle logo features and then complete the VLD task. Le Huan et al. [31] proposed a three-stage VLD method. First, they use the positional correlation between the license plate and the logo to coarsely position the vehicle logo. Then, they introduced the optimized Hough transform into the task of detecting the typical shape of the vehicle logo. Finally, a deep belief network is used to classify the vehicle logo. Huang et al. [32] integrated VGG-16 and ResNet-50 as feature extraction networks into Faster RCNN. They proved that the deeper network may not be better for different recognition tasks with different amounts of data. In reference [33], a method based on CNN is introduced to complete the task of vehicle logo detection. However, this method relies too much on prior knowledge between the vehicle logo and the license plate but this knowledge is not stable. In [34], the author introduced the CNN and whitening transformation technology into the VLD task. Chen et al. [35] proposed an image recognition framework with a capsule network and they proved that the framework is superior to CNNs. Huang et al. [36] introduced the inception architecture into the VLD task and built a deep CNN network to detect the vehicle logo. Xia et al. [37] proposed a method to recognize vehicle logos by combining CNN and multi-task learning. Soon et al. [38] proposed a new vehicle logo detection method; their method uses particle swarm optimization to select the CNN architecture and super parameters, and then fine-tune and train CNN. Zhang et al. [39] proposed a method for identifying vehicle logos. This method used a multi-scale parallel convolutional neural network for the VLD task. This method utilizes a multi-scale convolution kernel to extract features from the original data in a parallel way. In [40], the author introduced a SIFT descriptor of the interior structure and back-propagation neural network into the VLD task. Their method combines the Top-Hat transform with the shape descriptor to locate the vehicle logo from the image. In addition, they use the back-propagation neural network to recognize the vehicle logo. Thubsaeng [41] et al. proposed a two-stage VLD method. This method used CNN and gradient histogram pyramid features to complete the VLD task. In the first stage, they used CNN to detect candidate regions and identify vehicle logos. In the next stage, a PHOG with a Support Vector Machine classifier was used to verify the results of the first stage. Pan [42] et al. proposed a vehicle logo recognition method based on CNN. Mao et al. [43] proposed a CNN-based vehicle logo detection method. They also designed a dataset called VL to train CNN and test the performance of the system. Yang et al. [44] introduced a VLD method that uses a CNN-based detection network and hard example training in vehicle logo detection. Li et al. [45] proposed a CNN method based on MapReduce. Additionally, they introduced a new genetic method of global optimization and the Bayesian regularization method into the task of initializing the classifier weight. In reference [46], a vehicle logo super-resolution method based on Canonical Correlation Analysis (CCA) is proposed to facilitate the identification of vehicle logos. Shuo Yang et al. [47] propose a new multi-class VLD dataset, called VLD-45 (Vehicle Logo Dataset), which contains 45,000 images and 50,359 objects from 45 categories. The dataset uses six detectors and six existing classifiers to evaluate and show the baseline performance. Zhang et al. [48] propose a lightweight network structure with separable convolution to improve the real-time character of vehicle logos while implementing the method in embedded devices. Surwase et al. [49] propose a multi-scale multi-stream deep network for vehicle logo recognition and process the input vehicle logo image through each of the multi-scale streams to extract the robust features followed by the logo recognition module. The proposed network adopts a knowledge sharing strategy by enabling the sharing of learned features at each stream throughout the network. Shi et al. [50] proposed an intersection over the average loss for enhancing the bounding box regression. The method accelerates the convergence of bounding box regression than using the intersection over union loss.

In a traffic environment, background information is inevitably introduced, which may interfere with the VLD task. Additionally, the size of the vehicle logo is often very small, making VLD difficult. To address these challenges, our paper proposes a VLD method based on the YOLO-T model and the correlation of the vehicle space structure.

## 3. Framework of Proposed Method

### 3.1. Overall Framework of the Proposed VLD Method

In recent years, thanks to the collective efforts of researchers, vehicle logo detection technology has made remarkable progress. As a contemporary hot topic, deep learning technology is also extensively utilized in the field of image processing. To a certain extent, this has promoted the development process of vehicle logo detection technology. In recent years, some excellent object detection methods have been proposed one after another, such as Faster-RCNN (ZF) [51], SSD [52], and YOLOv3 [53]. Over the years, the YOLO series has become the de facto industry-level standard for efficient object detection. The growth of the YOLO community has greatly enriched its use on numerous hardware platforms and rich scenarios. The current YOLOv6 [54], -v7 [55], and -v8 all draw heavily on recent ideas in network design, training strategies, testing techniques, quantification, and optimization methods. In addition, YOLOv7 is applied to the Camellia oleifera fruit detection [56].

The further application of deep learning technology in the field of vehicle logo detection will greatly promote its development. Despite the significant progress made in vehicle logo detection technology, there is still a long way to go before it can be widely employed. False detection is a prevalent issue in detection tasks, and VLD tasks are no exception. False detection can have a significant impact on the extraction, analysis, and traffic flow statistics of vehicle information. Therefore, it is crucial to address the problem of false detection. To enhance detection accuracy and tackle this challenge, we propose a new method of vehicle logo detection based on deep learning and utilize the correlation between vehicle components. YOLOv3 presents great advantages in terms of speed and accuracy. Compared with the network structure of YOLOv3, the YOLOv3-SPP version adds an SPP module, showing better performance. However, when the input of YOLOv3-SPP is 608 × 608, the feature map sizes of the three detection structures are 19 × 19, 38 × 38, and 76 × 76. In the experiment, we found that the detection structures of 19 × 19 and 38 × 38 are sufficient to achieve good detection results in the task of vehicle logo detection. The 76 × 76 detection structure is a kind of redundancy. Based on the above analysis, we reconstruct YOLOv3-SPP and propose YOLO-T. The focus of this study is to improve the accuracy in the VLD task. Subsequent experiments have shown that our model’s accuracy exceeds that of the original structure of YOLOv3. The experimental results will be presented in the following section.

A vehicle is an integrated entity comprised of many different components, each with a strong positional relationship. In the case of vehicle logos, various parts of the vehicle possess a spatial position relationship with it, such as vehicle lights, license plates, wheels, and more. Therefore, some existing vehicle logo detection methods ingeniously use the correlation between vehicle components and logos. This involves two steps: first, detecting license plates and roughly locating the vehicle logo using the relationship between the two; second, accurately detecting the vehicle logo from the roughly located image. This method reduces the detection area and minimizes the adverse effects of background interference. Nevertheless, it cannot achieve favorable detection results in instances of missing license plates or poor visibility. Similarly, utilizing the positional relationship between the wheel and the vehicle logo to solve false detection is unreliable, as detecting wheels in low visibility conditions is also difficult. Conversely, vehicle lights have distinct advantages over license plates and wheels, namely: 1. The number of vehicle lights is greater than that of license plates and wheels; 2. Lights are easy to detect at different angles; 3. Even at night, vehicle lights remain highly visible.

**Figure 1:**
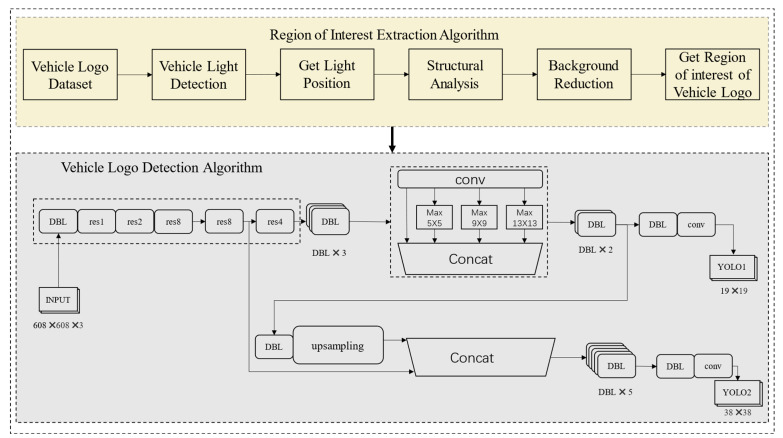
The overall framework of vehicle logo detection method.

In summary, the positional relationship between the vehicle light and the vehicle logo is more suitable for addressing false detection than the positional relationship between other components and the vehicle logo. We leverage the positional correlation between the vehicle light and the vehicle logo to solve the false detection issue and utilize YOLO-T to detect the vehicle logo. The frame chart of the proposed vehicle logo detection method is shown in Figure 1.

### 3.2. Spatial Correlation Analysis

Improving detection accuracy and avoiding false detection are crucial aspects of the VLD task. False detection can have a direct impact on the reliability of vehicle logo detection results. However, the traffic environment is highly complex and unpredictable, making it difficult to control. Thus, false detection is a difficult challenge in complex traffic environments. The primary objective of the VLD task is to detect the vehicle logo on the vehicle and extract relevant vehicle information. Billboards, characters, and external patterns on the vehicle are the primary causes of false detection. Therefore, eliminating the impact of these factors on VLD tasks is paramount in resolving the problem of error detection.

**Figure 2:**
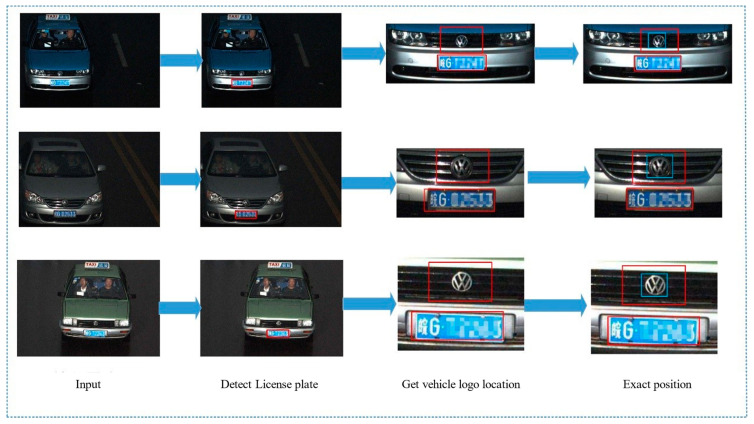
The flow chart of past vehicle logos detection.

In the VLD task, due to the influence of a complex environment, false detection is inevitable. At present, many vehicle logo detection methods [23,25,27,31,33] use the spatial relationship between the license plate and the vehicle logo to locate the vehicle logo. The general process is shown in Figure 2. First, the headlights are more numerous and can be easily detected even at different angles. Due to the characteristics of headlights, even at night and in fog and for other low visibility conditions, these are not difficult to detect. In real-world traffic conditions, the removal of headlights is rare. In addition, with more lights than license plates, detection is easier. Therefore, the constraint relationship between lights and logos is stronger than that between license plates and logos. In addition, visual measurement applications are applied to the Novel visual crack width measurement [57].

**Figure 3:**
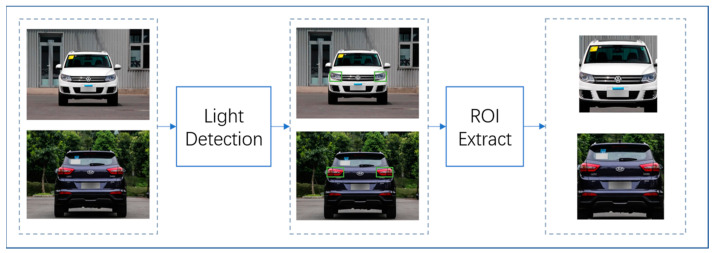
The flow chart of our method.

**Figure 4:**
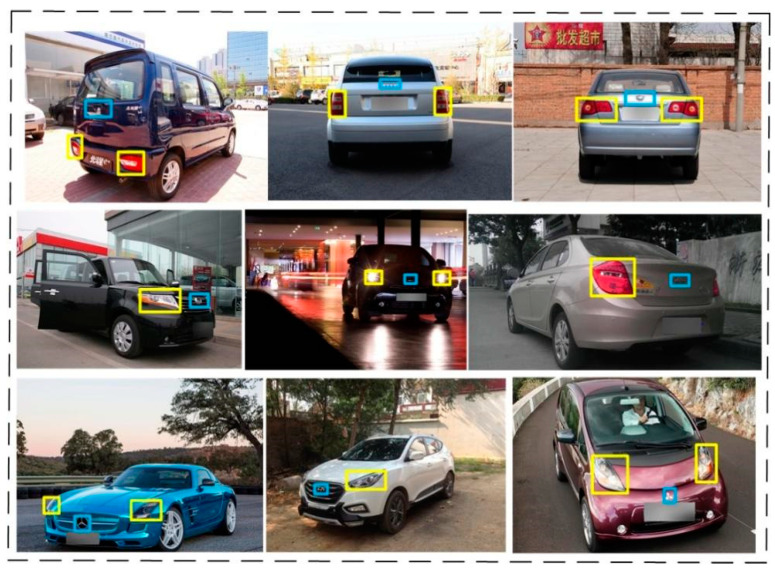
Examples of links between the vehicle logos and the vehicle lights. The yellow box denotes the location of the car lights while the blue box indicates the location of the car logo.

In this paper, we propose a method to use the spatial structure relevance of the vehicle to eliminate the impact of external environmental factors on the task of vehicle logo detection. Figure 3 shows the flow chart of our method. A vehicle is composed of many parts and there is a certain spatial positional relationship between them. Making full use of the positional correlation between the various components of a vehicle is not only helpful for coarse positioning but also helps to solve the problem of false detection. After repeated research analysis, we found that the positional correlation between the vehicle light and the vehicle logo is more reliable than the correlation between other components and the vehicle logo. In general, the vehicle logo is in a position parallel to the lights. However, in some special models, the relative positional relationship between the vehicle logo and the lights may vary. Figure 4 shows the various positional relationships between the logo and the lights. To demonstrate the correlation between the vehicle light position and the vehicle logo more vividly, we use a yellow box and a blue box to show their positions, respectively. The diversity of the positional relationship between the vehicle logos and the vehicle lights makes it challenging to retain vehicle logo information and eliminate background interference. Setting the size of the region of interest (ROI) is crucial in solving this issue. If the ROI is set too small, the logo information may be lost, which will have a fatal impact on the VLD task. Therefore, we should extract a rough area. When both headlights are detected, the left and right boundaries of the ROI are taken as the left and right boundary values of the coordinates of the headlights. The upper and lower boundaries are expanded by 150 pixels from the original drawing’s upper and lower boundaries. If it exceeds the original boundaries, we use the upper and lower boundaries of the original drawing as the boundary. When a single headlight is detected, the left and right edges of the original image are taken, and the upper and lower edges of the headlight are expanded by 150 pixels. If it exceeds the original boundaries, we use the upper and lower boundaries of the original drawing as the boundary. Since some car tags are far above or below the headlights, a larger area is taken to avoid extraction failure. The strategy of extracting the region of interest of the vehicle logo not only achieves the effect of coarse positioning of the vehicle logo but also weakens the effect of background interference.

### 3.3. The Structure of YOLO-T and Pre-Training Strategy

**Figure 5:**
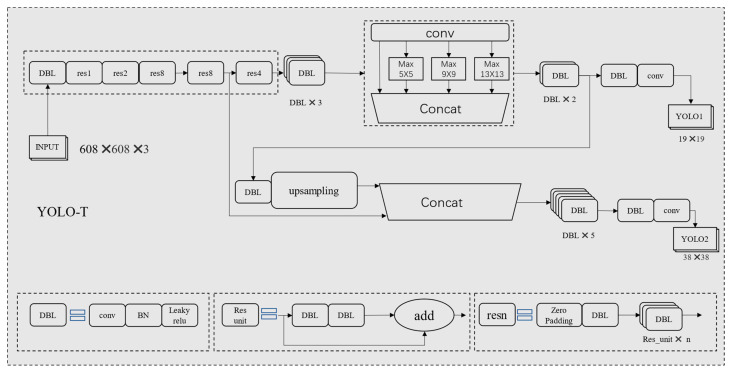
Architecture of the YOLO-T for VLD.

Compared to other traffic participants, the resolution of the vehicle logo itself is often lower. Additionally, the shooting angle and distance can alter the shape and size of the vehicle logo in the image. Detecting a small vehicle logo is an extremely challenging task, particularly in a complex background. As a result, improving detection ability is crucial to promoting the development of VLD technology. Fast R-CNN uses the last single convolutional layer to complete the task of object detection. SSD creatively adopts the method of combining shallow feature maps and deep feature maps for detection. However, neither Fast R-CNN nor SSD effectively fuses deep feature maps with shallow feature maps for object detection. In Convolutional Neural Networks (CNN), deep feature maps and shallow feature maps have different information focuses. The former is rich in location information, and the latter is rich in semantic information. Therefore, the effective fusion of shallow feature maps and deep feature maps helps to improve the performance of the network in target detection tasks. YOLOv3 has adopted this strategy and has shown good detection results; however, the task of vehicle identification detection needs to be adjusted. The SPP module is introduced in YOLOv3-SPP to improve the detection effect; however, during the experiment on vehicle logo detection, we found that this algorithm will lead to redundancy. During the experiment, we found that 19 × 19 and 38 × 38 detection structures are sufficient to obtain good results. Therefore, the 76 × 76 network structure is actually redundant. In response to the above shortcomings, we propose a new detection network, YOLO-T. The network structure of YOLO-T is shown in Figure 5. The hyperparameter optimization method of model setting is mainly for the adjustment of the learning rate. During training, 16 samples are treated iteratively in one round, and BN (batch normalization) is used in each weight update. Decay is set to 0.005, Momentum is set to 0.9, and the initial learning rate is set to 0.001. In order to make the model iteration relatively stable in the initial iteration stage, a learning rate change point burn_in is set as 1000. In the first 1000 rounds of iteration, the learning rate calculation formula is as follows:

(1) $$\mathrm{Learn}\_\mathrm{rate}=\mathrm{last}\_\mathrm{learn}\_\mathrm{rate}\;\times {\left(\frac{\mathrm{batch}\_\mathrm{num}}{\mathrm{burn}\_\mathrm{in}}\right)}^{0.95}$$

At 40,000 iterations, the learning rate will decline 10 times, and at 45,000 iterations, the learning rate will decline 10 times on the basis of the previous learning rate.

DBL refers to the DarknetConv2D_BN_Leaky convolutional layer, comprising of a 2D convolutional layer in Darknet, namely DarknetConv2D; a batch normalization layer, used to normalize input data x, namely BatchNormalization(); and a LeakyReLU layer with a slope of 0.1.DarknetConv2D is a 2D convolution in Darknet. Regularize the kernel weight matrix using L2 regularization with a parameter of 5 × 10^−4^ to regularize the kernel weight parameters w. Padding is usually done in the same mode. Only when the stride is (2, 2), use the valid mode. This approach helps avoid the introduction of meaningless boundary information during downsampling. The remaining parameters remain consistent with the 2D convolution operation, Conv2D(). The res unit relates to the residual component, which comprises of two convolutional layers and a shortcut link. This structure can be used to make the network structure deeper. In the resn structure, n is the number that tells you how many res_units are in this res_block. This structure includes zero-padding and residual structure, as shown in the bottom right corner of the figure above. Concat: The tensors amalgamate with one another, achieving the goal of multi-scale feature fusion. It is important to note that the operation of concatenation differs from that of the residual layer addition. Concatenation will expand the dimension of the tensor, while add just adds directly without changing the dimension of the tensor. Max n × n represents the kernel size n × n. The whole module draws on the idea of spatial pyramid pooling. After the feature map is fused with local features and global features, the expressive ability of the feature map is enriched, which is beneficial to the situation where the target size difference in the image to be detected is large, and the detection accuracy is improved.

In the task of object detection, it is very important to pre-train the detection network. In many training tasks, the original classification model of the network is used as a pre-training model to initialize the parameter settings of the training detector. In this article, we adopted a new pre-training method to improve the accuracy of vehicle logo detection tasks. Specifically, we use the vehicle logo images processed by CycleGAN [58] to train the network. CycleGAN can make images appear in different styles, thereby enhancing the diversity of the dataset. After training the network through this training strategy, the weight of the previous part is intercepted as a pre-training model. Then, the weights of the network are initialized by the pre-trained model.

## 4. Experiments

### 4.1. LOGO-17 Dataset

**Figure 6:**
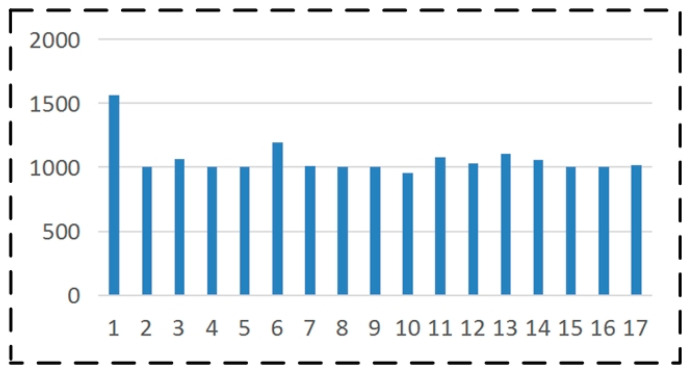
Display and comparison of the number of each type of vehicle logo in LOGO-17 dataset.

The development of vehicle logo detection technology is constrained by multiple factors and the lack of datasets presents a significant challenge. Convolutional neural networks require large amounts of data to learn features and obtain robust models. However, as per our research, there is currently no extensive open-source vehicle logo dataset available, which hinders the verification of many algorithms and impedes the development of vehicle logo detection. Considering these reasons, we have compiled a LOGO-17 vehicle label dataset. The vehicle logo dataset consists of 18,089 images from 17 different types of vehicle logos. To visually represent the quantity of each category, we display the number of various vehicle logos in LOGO-17 in the form of a histogram in Figure 6.

**Figure 7:**
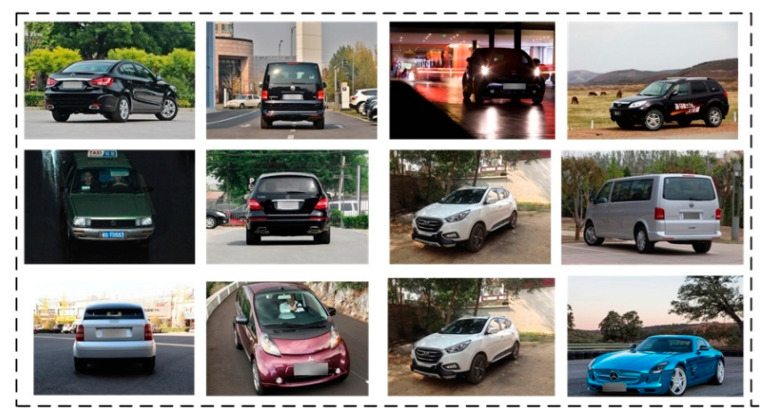
Some images in the LOGO-17 dataset.

The images contained in the LOGO-17 dataset exhibit a broad range of diversity with respect to shooting angles, backgrounds, and pixel sizes, thereby closely simulating real-world scenarios. It is noteworthy that the majority of images within the dataset do not include license plates. In Figure 7, we present a selection of images from the LOGO-17 dataset. Presently, many methods for vehicle logo detection rely on the spatial relationship between the license plate and the vehicle logo in order to locate the latter. However, in practical situations, license plates are frequently absent, damaged, or obstructed. Our research has taken this issue into consideration.

As for the computer platform settings, a standard PC was used for all the experiments, whose hardware and software configuration are listed as follows:

- NVIDIA Quadro RTX 4000 GPU;
- Dynamic Memory: 64G DDR4 RAM;
- Ubuntu 20.04.3 LTS.

### 4.2. Experiment Results

In order to verify the superiority of our proposed method, we compare it with other detection methods. The experimental results are shown in Table 1. Our method has high detection accuracy. The best result, we bolded it in the table.

**Figure 8:**
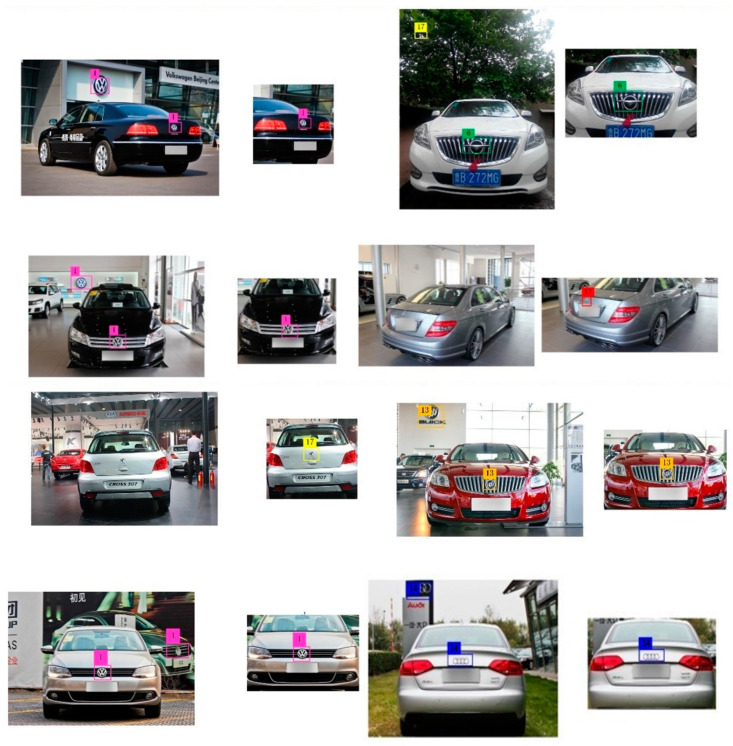
The effect contrast of the region of interest was extracted.

The detection effect is shown in Figure 8. Under the action of the region of interest extraction algorithm, the number of false detections of vehicle signs caused by background interference is effectively reduced, and some undetected vehicle signs are easier to be detected. There is no best model, only the one that best fits your data set. The deepening of the model does not necessarily make the effect better. The multiple model parameters may also have a large number of redundant parameters for the task of vehicle logo detection. Table 2 shows the performance of our model, the following experiments show that our model has improved for the VLD task. 

## 5. Conclusions and Future Works

In recent years, the YOLO series has emerged constantly and updated continuously, already up to version 8. However, the effectiveness of a series cannot be simply judged by its version number. Different versions of YOLO have their own characteristics and can be chosen based on actual needs in different scenarios, upstream and downstream environments, and resource support situations. There is no best model, only the one that best fits your dataset. The deepening of the model does not necessarily make the effect better.

This paper proposed a new method of VLD based on the spatial correlation of the vehicle and YOLO-T network. We combine lamp detection with vehicle logo detection to reduce the interference of the external environment to the vehicle logo detection task. After obtaining the position of the vehicle lamps, the areas where the vehicle logo is unlikely to appear are excluded according to the spatial position correlation of the vehicle lamps and the vehicle logo. YOLO-T network uses the combination of a top-down and bottom-up pyramid network structure to enhance the path and uses the multi-scale method to detect. This method can not only improve the accuracy of vehicle logo detection but also reduce the interference of the surrounding environment to a vehicle logo detection task. Based on the experimental results, our method is more effective than other methods in vehicle logo detection.

Although many researchers have been deeply involved in the field of vehicle logo detection for many years, there is still a long way to go before the application of vehicle logo detection technology. In the future, we may further introduce deep learning technology into the vehicle logo detection task, so as to further improve the vehicle logo detection effect and promote the development of vehicle logo detection technology. In the future, it is necessary to explore new methods to further improve the detection capability of VLD tasks. This is a direction we are currently pursuing.

## Figures and Tables

**Table 1:** The Comparison of VLD Results Using Different Methods for the LOGO-17 Dataset.

Number		Method	Method [32]	Method [44]	Method [51]	Method [52]	YOLOV3	YOLOV7	YOLOV8	Our Method
	Manufacturer									
1	VOLKSWAGEN		88.5	93.3	89.0	89.5	92.9	94.5	**98.2**	97.2
2	BYD		83.7	90.5	82.1	88.2	83.9	93.2	96.0	**98.0**
3	First Automobile Works		84.9	90.8	83.1	89.6	94.0	96.4	96.9	**99.3**
4	MITSUBISHI		77.6	88.0	62.4	85.3	89.7	93.6	96.8	**97.3**
5	Hyundai		81.2	85.8	75.3	88.9	87.6	93.0	95.8	**98.3**
6	HAIMA		89.9	91.6	86.7	89.0	96.3	90.5	98.1	**99.6**
7	GREAT WALL		80.8	92.7	72.1	93.6	93.9	90.4	98.5	**99.9**
8	SUZUKI		83.6	93.9	66.4	90.2	96.0	87.5	96.8	**97.0**
9	KIA		87.1	97.8	84.6	93.2	97.4	97.8	98.8	**99.9**
10	ZHONGHUA		87.3	95.8	80.8	92.6	96.8	98.2	98.2	**99.9**
11	TOYOTA		83.5	86.9	67.7	90.4	88.4	95.6	96.4	**98.8**
12	BENZ		85.9	92.8	77.1	89.6	94.4	95.1	97.8	**97.6**
13	BUICK		88.4	97.4	80.5	95.7	97.3	98.4	98.6	**99.2**
14	AUDI		90.0	98.4	85.6	89.7	98.7	91.5	**99.5**	99.1
15	CHERY		86.2	86.7	73.3	88.8	85.7	89.1	93.1	**98.9**
16	JAC		76.7	82.7	73.9	87.5	81.8	89.6	94.9	**99.0**
17	PEUGEOT		81.0	89.7	70.9	85.4	94.0	93.8	**96.9**	95.8
Mean Average Precision			84.4	91.4	77.1	89.8	92.2	93.7	97.1	**98.5**

**Table 2:** Structure and results of ablation experimental model.

Experiment	Method	Mean Average Precision
1	YOLOV3	92.2
2	YOLOV3 + YOLO-T	95.8 (+3.6)
3	YOLOV7	93.7
4	YOLOV7 + YOLO-T	98.4 (+4.7)
5	YOLOV8	97.1
6	YOLOV8 + YOLO-T	99.0 (+1.9)

## Data Availability

Not applicable.
